# Alkaloid biosynthesis in medicinal crop kratom (*Mitragyna speciosa*) varies with postharvest, genetic, and seasonal factors

**DOI:** 10.3389/fpls.2025.1653916

**Published:** 2025-09-29

**Authors:** Mengzi Zhang, Annabella Lyndon, Siva Rama Raju Kanumuri, Abhisheak Sharma, Brian J. Pearson, Christopher R. McCurdy, Jianjun Chen

**Affiliations:** ^1^ Environmental Horticulture Department, Mid-Florida Research and Education Center, University of Florida, Apopka, FL, United States; ^2^ Department of Pharmaceutics, College of Pharmacy, University of Florida, Gainesville, FL, United States; ^3^ Translational Drug Development Core, Clinical and Translational Science Institute, University of Florida, Gainesville, FL, United States; ^4^ Mid-Columbia Agricultural Research and Extension Center, College of Agricultural Sciences, Oregon State University, Hood River, OR, United States; ^5^ Department of Medicinal Chemistry, College of Pharmacy, University of Florida, Gainesville, FL, United States

**Keywords:** drying temperature, withering, indole alkaloid, mitragynine, 7-hydroxymitragynine, speciogynine

## Abstract

**Introduction:**

Kratom (*Mitragyna speciosa*), a medicinally valuable ethnobotanical tree native to Southeast Asia, has traditionally been used to combat fatigue and enhance productivity. Recently, it has gained attention in North America and Europe for its potential therapeutic applications, particularly in pain management and opioid withdrawal, positioning it as a candidate for drug development. Postharvest processing is a critical stage that influences chemical transformations of bioactive compounds, yet its impact on kratom remains poorly understood.

**Methods:**

This study investigated the effects of withering duration, drying temperature, cultivar, and season on kratom alkaloid composition and concentration. In Study I, leaves of cultivar Hawaii underwent four withering durations (0, 12, 24, 72 h) followed by drying at five temperatures (−40, 25, 40, 60, 80 °C). In Study II, leaves of cultivar MR-Malaysian were tested under two withering durations (0, 12 h) and two drying temperatures (25, 60 °C). Both studies were conducted across two seasons.

**Results:**

Withering generally increased mitragynine concentrations by 14-65% (w/w) in 'Hawaii' and 3-8% in 'MR-Malaysian' in leaf alkaloid extracts. A 12-h withering followed by drying below 40 °C enhanced speciogynine and paynantheine in 'Hawaii' by 37-48% and 35-67%, respectively. Low drying temperatures preserved mitragynine, speciogynine, and paynantheine across cultivars. The average 7-hydroxymitragynine content in leaf alkaloid extracts ranged from 0.02-0.04% and was detected only in specific seasons, varying by cultivar, suggesting genotype-environment interactions.

**Discussion:**

This study demonstrates for the first time that kratom alkaloid composition and concentration are substantially influenced by genotype, season, and postharvest handling. These findings underscore the importance of optimizing postharvest processing strategies to enhance beneficial alkaloid profiles in kratom.

## 1 Introduction

**Figure 1:**
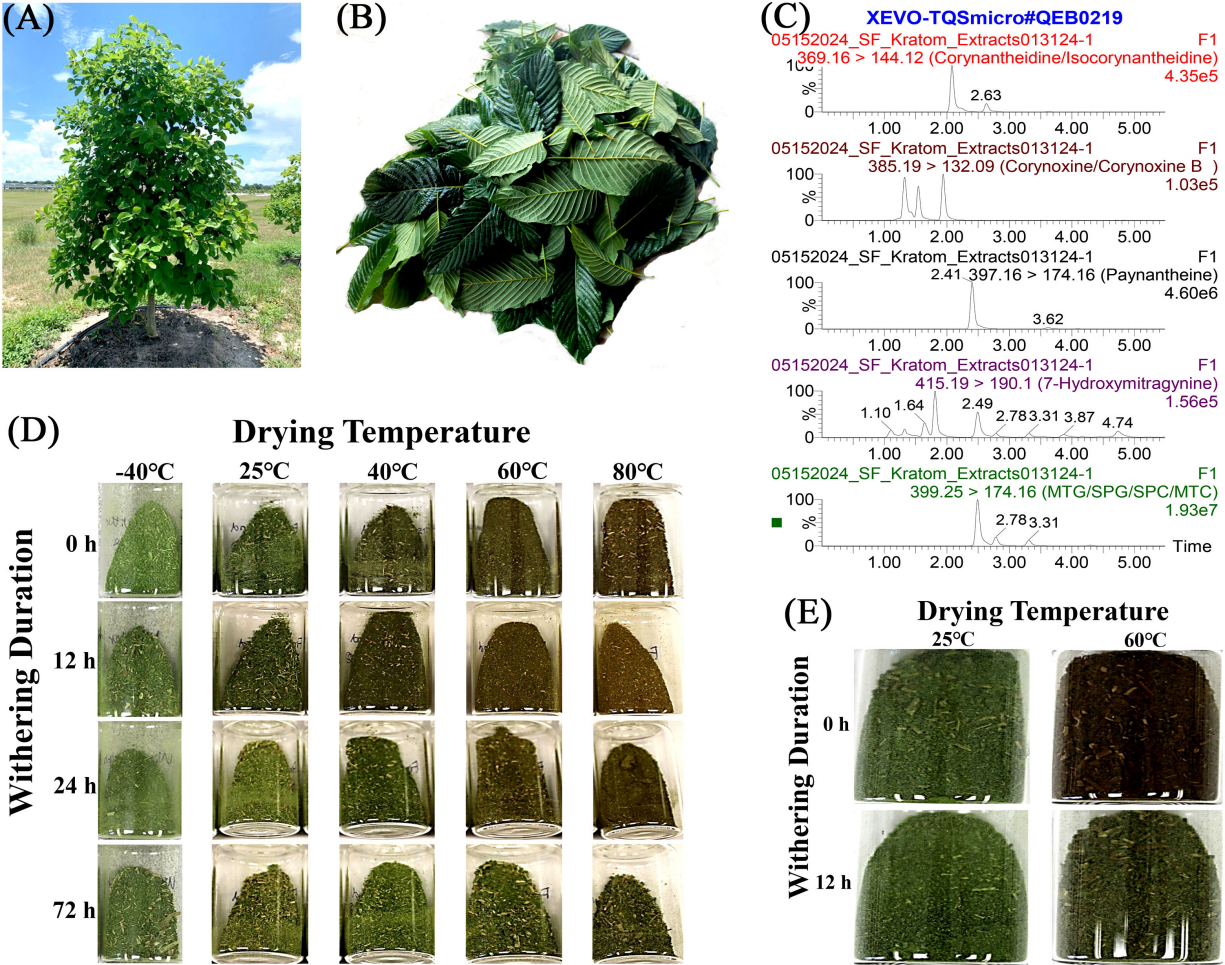
A process of postharvest treatments on collected kratom leaves for analysis of alkaloids. **(A)** Kratom tree; **(B)** Harvested bulk kratom leaves; **(C)** Representative LC-MS/MS chromatogram of sample #013124-1; **(D)** Powder color of cultivar Hawaii under each postharvest treatment from Study I; **(E)** Powder color of cultivar MR-Malaysian under each postharvest treatment from Study II.

Kratom (*Mitragyna* sp*eciosa* Korth), a small-to-medium sized tropical tree indigenous to Southeast Asia (**Figure 1A**), has been consumed for centuries by native people as an ethnobotanical medicine to relieve pain and fatigue, treat diarrhea, enhance work productivity, and assist in opioid tapering and withdrawal alleviation (Suwanlert, 1975; Cinosi et al., 2015; Grundmann, 2017). The pharmacological properties of kratom are derived from indole alkaloids that induce non-opioid (dose-dependent stimulant properties) and opioid-like effects upon consumption (Grundmann et al., 2023). More than 40 monoterpene alkaloids have been identified in kratom, and a portion of those and their respective analogs are capable of binding opioid and adrenergic receptors throughout the body. The primary alkaloid, mitragynine, constitutes 0.7-38.7% of the alkaloid suite by mass and produces opioid-like antinociception effects upon binding µ-opioid receptors with partial agonistic activity (Váradi et al., 2016; Obeng et al., 2019). Its metabolite and structural analog, 7-hydroxymitragynine, possesses greater binding and functional activity at µ-opioid receptor than that of morphine; however, its concentration in nature is typically low (Horie et al., 2005; Hassan et al., 2013; Kruegel et al., 2016; Hemby et al., 2019; Obeng et al., 2019). Other minor alkaloids, such as paynantheine, speciogynine, and speciociliatine, possess similar serotonergic and adrenergic receptor binding (Obeng et al., 2019). Together, these alkaloids provide a comprehensive pharmacological effect obtained when consuming kratom products (León et al., 2021; Grundmann et al., 2023).

Over the past two decades, there has been a notable expansion of consumer interest in kratom beyond Asian borders into North America and Europe. Recent surveys indicated that 0.7-6.1% of the United States population was using kratom products, and the use was more prevalent among those with prior substance use disorder (Palamar, 2021; McCurdy et al., 2024). In Western countries, kratom is most commonly available as dried, ground leaf powder, oftentimes encapsulated or occasionally tableted for ease of consumption (Grundmann, 2017). Liquid extracts, resins, and soft gels usually contain high or sometimes excessive alkaloid concentrations. Such products are easily obtainable in North American markets (Kamble et al., 2021; Sharma et al., 2025). Contrarily, in Southeast Asia, kratom is primarily consumed as freshly harvested leaves by chewing directly or brewing in boiling water for 2-3 hours as a tea decoction. However, processed kratom products, like harvested leaves, crumbs, and powders, are also available in these regions (Suwanlert, 1975; Syarma et al., 2019).

The harvest and postharvest processing of kratom leaves are largely undocumented in the literature. Information gathered through the internet suggested that kratom is typically dried either directly after leaf harvesting, sorting, and washing or after leaf fermentation in Southeast Asia. Environmentally, kratom leaves can be dried either indoors, outdoors, or with a combination of both. Indoor drying avoids direct sunlight exposure and oftentimes takes place on shelved racks in darkened rooms equipped with fans to circulate air and accelerate evaporation. Outdoor drying is achieved by spreading the leaves on a sheet under direct sunlight where they are exposed to higher heat and stronger light. It is believed that the quality of the kratom product vary with different postharvest methods, thus resulting in different alkaloid profiles.

Contradictory results have been reported on the effect of drying temperatures on alkaloid concentration in other plant species. For example, Ademiluyi et al. (2018) suggested that the alkaloid in Moringa (*Moringa oleifera*) leaf decreased by 63% and 21% when drying under the sun and oven (40°C), respectively, compared to air drying at room temperature. Similarly, canadine from the medicinal forest herb goldenseal (*Hydrastis canadensis*) significantly decreased when drying temperatures increased beyond 30°C and its level decreased by more than 50% when dried under 54.5°C compared to freeze-drying (Zuiderveen et al., 2021). In contrast, compared to air drying (22-32°C), caffeine concentration in *Ilex guayusa* leaves was similar when dried under the sun (~34°C) but increased by 23% in the oven (46°C) (Manzano Santana et al., 2018).

Withering is a natural wilting process of leaves after they are removed from the plant. This is a critical step in tea production due to the limited capacity of leaf processing at peak harvest seasons. Typically, freshly harvested tea leaves are spread out on withering racks in sheds until they can be further processed. During this time, the leaves become soft due to moisture loss, resulting in internal chemical changes that shape the final product's flavor, aroma, and appearance. Since kratom is commonly consumed similar to a tea product in Southeast Asia, this practice may have been implemented unconsciously in the kratom postharvest process in Southeast Asia, although this is undocumented. Withering conditions and methods can significantly affect chemical composition of plant products. For example, Jia et al. (2023) found that sunlight and trough withering – compared to charcoal fire withering, which differed in temperature, duration, and turning intervals – resulted in significantly higher alkaloid concentrations in Wuyi rock tea. In a separate study, withering tea leaves for 18 hours at 15°C, 20°C, and 25°C resulted in an increase in caffeine concentration by 16%, 9%, and 10%, respectively, when compared to the initial caffeine concentration (Ye et al., 2018).

Most domestically available commercial kratom products contain moderate concentrations of mitragynine and low concentrations of 7-hydroxymitragynine (Sharma et al., 2019, 2025). In contrast, anecdotally, our kratom leaf samples possessed comparable concentrations of mitragynine but no 7-hydroxymitragynine, suggesting that the production of 7-hydroxymitragynine may be through postharvest processes. Thus, a better understanding of the postharvest environmental influence on kratom product quality is needed. The objective of this study was to assess the impact of postharvest drying temperatures and withering durations on the accumulation of fourteen alkaloids in leaves of two kratom cultivars harvested in two different seasons.

## 2 Materials and methods

### 2.1 Study I

#### 2.1.1 Leaf materials

Bulk fresh leaves were harvested from ‘Hawaii’ kratom mother stocks (**Figure 1A**) on July 12 (hereafter referred to as summer) and November 15 (hereafter referred to as winter) 2022, respectively. Healthy, fresh, mature leaves (**Figure 1B**) were collected from similar positions on branches (2nd or 3rd pair of leaves from the meristem) throughout the trees to ensure uniform maturity and photosynthetic activity, thereby reducing potential variability in alkaloid concentration. Harvested leaves were placed into a collection bucket and mixed thoroughly before being randomly assigned to each postharvest treatment. Details on the maintenance and management of the ‘Hawaii’ mother stock are provided in the **Supplementary Information**.

#### 2.1.2 Postharvest treatment and environmental control

Harvested composite bulk leaves were evenly divided into 48 samples for the summer harvest and 60 samples for the winter harvest. All samples were randomly assigned to one of four withering durations (0, 12, 24, and 72 hours). Leaves were laid flat in a single layer on air filters and placed in environmentally controlled withering rooms for the assigned durations. Each withering room was identical and equipped with an individual air conditioning unit. Air temperature and relative humidity were monitored and recorded using a data logger (Elitech, San Jose, CA, United States) at 5-minute intervals. The withering rooms maintained an average air temperature of 25°C and a relative humidity of 55%, with no supplemental lighting.

Following withering, leaf samples from each withering duration group were further divided into four portions and subjected to drying treatments at either 25, 40, 60, or 80°C. A 25°C drying treatment was conducted using a forced air fan system within environmentally controlled rooms for six days, with an average air temperature of 25°C. The 40, 60, and 80°C drying treatments were achieved using thermo-controlled drying ovens (40GC, Qincy Lab Inc., Burr Ridge, IL, United States; SWN-46-6E, Wisconsin Oven Corp., East Troy, WI, United States; 4CEM, Precision Scientific Group, Chicago, IL, United States) for five days. For winter-harvested leaves, a freeze-drying treatment at -40°C was added as the fifth drying temperature. The freeze-drying treatment was performed using a freeze dryer (Harvest Right, Salt Lake City, Utah, United States). Detailed descriptions of all treatment combinations are listed in **Table 1**. Each treatment in both the summer and winter experiments included three replicate composite leaf samples.

**Table 1:** Summary of postharvest treatments used in Study I for cultivar Hawaii.

Study	Treatment	Withering duration	Drying temperature
I	1	0 hour	-40°C (only for winter harvest)
	2	0 hour	25°C
	3	0 hour	40°C
	4	0 hour	60°C
	5	0 hour	80°C
	6	12 hours	-40°C (only for winter harvest)
	7	12 hours	25°C
	8	12 hours	40°C
	9	12 hours	60°C
	10	12 hours	80°C
	11	24 hours	-40°C (only for winter harvest)
	12	24 hours	25°C
	13	24 hours	40°C
	14	24 hours	60°C
	15	24 hours	80°C
	16	72 hours	-40°C (only for winter harvest)
	17	72 hours	25°C
	18	72 hours	40°C
	19	72 hours	60°C
	20	72 hours	80°C

### 2.2 Study II

#### 2.2.1 Leaf materials

A different kratom cultivar, Mitragynine-Rich (MR)-Malaysian, was used for this study. As in Study I, healthy, fresh, mature leaves were harvested from ‘MR-Malaysian’ kratom mother stocks, using the second or third pair of leaves from the apical meristem to ensure maturity and consistency. The harvested leaves were thoroughly mixed and randomly assigned to post-harvest treatments. Leaves were harvested twice in 2024: January 25 (hereafter referred to as winter) and June 12 (hereafter referred to as summer). Details on the maintenance and management of the ‘MR-Malaysian’ mother stock are provided in the **Supplementary Information**.

#### 2.2.2 Postharvest treatment and environmental control

Similarly to Study I, harvested composite leaves were mixed thoroughly and evenly divided into 40 samples. All samples were subjected to one of two withering durations (0 or 12 hours). After withering, each group was further divided into two portions and dried at either 25°C or 60°C, with 10 leaf samples in each portion. A detailed description of the treatment combinations is provided in **Table 2**. The treatment setup was similar to Study I, except that a 25°C drying temperature was achieved using a food dehydrator (Weston PRO-2400, Weston, FL, United States) with a maximum temperature setpoint of 25°C.

**Table 2:** Summary of postharvest treatments used in Study II for cultivar MR-Malaysian.

Study	Treatment	Withering duration	Drying temperature
II	1	0 hour	25°C
	2	0 hour	60°C
	3	12 hours	25°C
	4	12 hours	60°C

### 2.3 Leaf alkaloid extraction and quantification

For both Study I and II, dried kratom leaves from each postharvest treatment were collected and ground into fine powders using a commercial grinder (BCG211, KitchenAid, St. Joseph, MI, United States). Subsequently, leaf alkaloids were extracted using the protocol described by Zhang et al. (2020) with slight modifications. Briefly, 0.5 g of fine-grinded kratom leaf powder samples were extracted with 3 mL 190 proof ethanol under water-bath sonication until exhaustion. The process was replicated three times to maximize alkaloid extraction. Quantification of alkaloids was performed using a Waters Xevo TQ-S Micro triple quadrupole mass spectrometer detector connected to Acquity Class I ultra performance liquid chromatography (UPLC) (Milford, MA, United States) and the chromatogram of a representative sample is provided in **Figure 1C**. UPLC method, compound, and source parameters were the same as reported previously (Zhang et al., 2020). Alkaloid content (% w/w) was reported based on leaf extracts, as postharvest treatments can potentially affect extraction efficiency, making extract-based values more accurate.

### 2.4 Experiment design and statistical analysis

Both Study I and Study II were conducted using a completely randomized design, with three replications in Study I and 10 replications in Study II. Statistical analysis was performed using a restricted maximum likelihood mixed model analysis in JMP Pro 16 (SAS Institute, Inc., Cary, NC, United States). Unless otherwise noted, *post-hoc* mean separation tests were performed on values above lower limit of quantification (LLOQ) using Tukey’s honest significant difference test. Statistical tests were considered significant if P ≤ 0.05.

## 3 Results

### 3.1 Powder color

Kratom powder presented a reddish-brown color under high drying temperatures compared to a green color under -40°C and 25°C (**Figures 1D, E**). The color change was more substantial when the withering duration was short, such as 0 or 12 hours. Increasing the withering duration up to 72 hours preserved the greenness of the powder, especially under high drying temperatures.

### 3.2 Effects of withering duration on bioactive compounds

**Figure 2:**
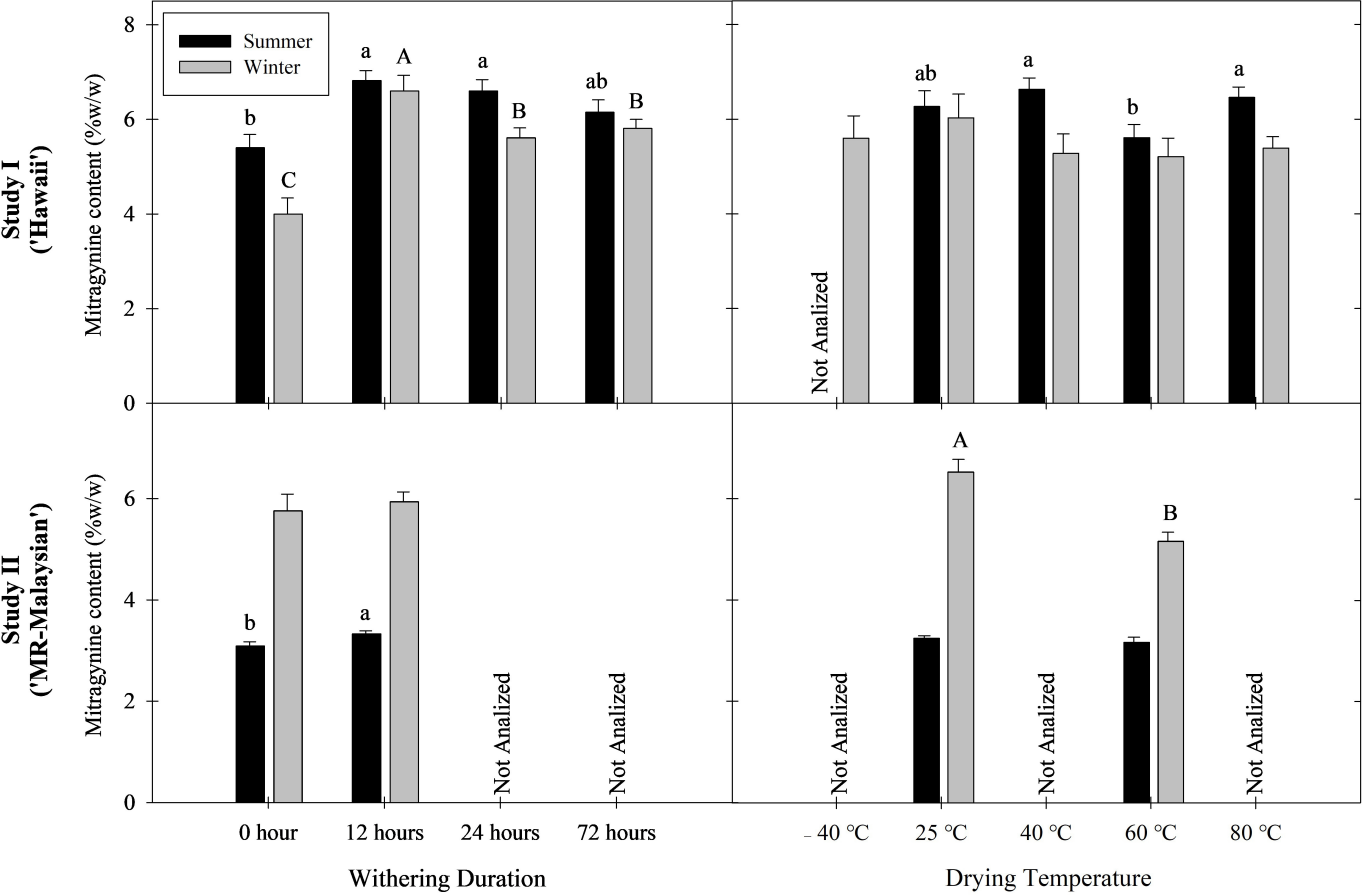
Mitragynine content in leaf alkaloid extracts across treatments in Study I (cultivar Hawaii) and Study II (cultivar MR-Malaysian). Lowercase letters denote summer treatment differences; uppercase letters denote winter differences. Treatments with different letters are significantly different according to Tukey’s HSD test (Study I) and Student’s t-test (Study II) at P < 0.05.

To facilitate interpretation of the results, the treatment without withering followed by drying at 25°C was considered as the baseline (control). Withering significantly increased mitragynine concentration in both studies. Overall, mitragynine concentration was elevated by 14-65% in ‘Hawaii’ leaf extract and 3-8% in ‘MR-Malaysian’ extract (**Figure 2**). Specifically, a 12-hour withering period followed by drying at -40, 25, 40, and 60°C increased mitragynine concentration by 117%, 38-123%, 16-61%, and 43-103% in ‘Hawaii’, respectively (**Table 3**). Extending the duration of withering from 12 to 24 and 72 hours did not further change the mitragynine concentration compared to the 12-hour treatment. Still, the concentrations remained significantly higher, or at least comparable, to those observed in non-withered leaf samples. Interestingly, withering duration had minimal impact on mitragynine concentration in ‘MR-Malaysian’, except for those harvested in the summer and dried at 60°C (**Table 4**).

**Table 3:** Mitragynine content (% w/w ± _SE_) in leaf alkaloid extracts from Study I (cultivar Hawaii) under postharvest treatments by season.

Withering duration	Mitragynine (%w/w)				
	— 40°C	25°C	40°C	60°C	80°C
Summer					
0 hour	NA	4.87 ± 0.36 **bAB**	6.19 ± 0.24 **A**	4.34 ± 0.12 **bB**	6.22 ± 0.38 **abcA**
12 hours		6.72 ± 0.39 **a**	7.18 ± 0.58	6.22 ± 0.28 **a**	7.15 ± 0.27 **ab**
24 hours		7.38 ± 0.60 **a**	6.89 ± 0.31	5.87 ± 0.35 **ab**	6.25 ± 0.22 **abc**
72 hours		6.10 ± 0.32 **ab**	6.26 ± 0.67	6.00 ± 0.66 **ab**	6.24 ± 0.66 **abc**
Winter					
0 hour	3.43 ± 0.28 **cB**	3.60 ± 0.43 **cB**	3.58 ± 0.33 **bB**	3.17 ± 0.11 **bB**	6.19 ± 0.68 **A**
12 hours	7.47 ± 0.40 **aAB**	8.04 ± 0.34 **aA**	5.77 ± 0.74 **aBC**	6.42 ± 0.33 **aABC**	5.31 ± 0.42 **C**
24 hours	5.29 ± 0.24 **b**	6.14 ± 0.24 **b**	6.26 ± 0.66 **a**	5.43 ± 0.41 **a**	4.93 ± 0.37
72 hours	6.21 ± 0.46 **ab**	6.35 ± 0.16 **b**	5.51 ± 0.73 **a**	5.83 ± 0.06 **a**	5.15 ± 0.17

Mean comparisons were conducted within each season. Lowercase letters indicate mean separation for withering duration; uppercase letters indicate mean separation for drying temperature. Means sharing different letters are statistically different by Tukey’s HSD test at P < 0.05. NA = not analyzed.

**Table 4:** Key alkaloid content (% w/w ± _SE_) in leaf alkaloid extracts from Study II (cultivar MR-Malaysian) under postharvest treatments by season.

		Winter		Summer	
		25°C	60°C	25°C	60°C
Mitragynine	0 hour	6.67 ± 0.45 **a**	4.87 ± 0.26 **c**	3.25 ± 0.07 **a**	2.89 ± 0.14 **b**
	12 hours	6.41 ± 0.23 **ab**	5.47 ± 0.23 **bc**	3.26 ± 0.07 **ab**	3.39 ± 0.09 **a**
7-Hydroxymitragynine	0 hour	0.032 ± 0.001 **ab**	0.023 ± 0.001 **c**	Below LLOQ^x^	Below LLOQ
	12 hours	0.035 ± 0.001 **a**	0.029 ± 0.002 **bc**	Below LLOQ^y^	Below LLOQ
Speciogynine	0 hour	0.75 ± 0.03 **ab**	0.65 ± 0.03 **b**	0.45 ± 0.01 **b**	0.50 ± 0.01 **ab**
	12 hours	0.75 ± 0.03 **ab**	0.79 ± 0.04 **a**	0.44 ± 0.01 **b**	0.51 ± 0.02 **a**
Paynantheine	0 hour	1.04 ± 0.03 **a**	0.91 ± 0.02 **b**	0.65 ± 0.02	0.64 ± 0.02
	12 hours	1.01 ± 0.03 **ab**	1.02 ± 0.04 **ab**	0.64 ± 0.02	0.72 ± 0.02
Corynantheidine	0 hour	0.042 ± 0.005	0.038 ± 0.005	0.042 ± 0.003 **ab**	0.032 ± 0.005 **b**
	12 hours	0.045 ± 0.004	0.034 ± 0.004	0.052 ± 0.006 **a**	0.034 ± 0.002 **b**
Speciociliatine	0 hour	0.36 ± 0.03 **b**	0.43 ± 0.03 **ab**	0.55 ± 0.04	0.54 ± 0.03
	12 hours	0.46 ± 0.04 **ab**	0.52 ± 0.04 **a**	0.55 ± 0.05	0.65 ± 0.06
Mitraciliatine	0 hour	0.028 ± 0.003 **b**	0.033 ± 0.003 **ab**	0.054 ± 0.004	0.049 ± 0.004
	12 hours	0.045 ± 0.005 **a**	0.041 ± 0.004 **ab**	0.049 ± 0.006	0.059 ± 0.006
Isopaynatheine	0 hour	0.017 ± 0.002 **b**	0.019 ± 0.002 **ab**	0.028 ± 0.003	0.026 ± 0.002
	12 hours	0.027 ± 0.003 **a**	0.023 ± 0.002 **ab**	0.030 ± 0.006	0.031 ± 0.003
Corynoxine A	0 hour	0.053 ± 0.006 **a**	Below LLOQ	0.035 ± 0.003	0.045 ± 0.003
	12 hours	0.041 ± 0.004 **b**	Below LLOQ	0.040 ± 0.002	0.041 ± 0.003
Corynoxine B	0 hour	0.034 ± 0.005 **bc**	0.045 ± 0.005 **ab**	0.038 ± 0.003	0.049 ± 0.004
	12 hours	0.025 ± 0.003 **c**	0.056 ± 0.003 **a**	0.042 ± 0.002	0.040 ± 0.002
9-Hydroxycorynantheidine	0 hour	yes detection	no detection	NA	NA
	12 hours	yes detection	no detection	NA	NA

Mean comparisons were conducted separately for each alkaloid and season. Means sharing different letters are statistically different by Tukey’s HSD test at P < 0.05. NA, not analyzed. LLOQ, Lower Limit of Quantification (limit of quantification = 0.005% in leaf alkaloid extract). ^x^1 out of 10 samples were above LLOQ. ^y^2 out of 10 samples were above LLOQ.

For 7-hydroxymitragynine, a significant difference in the number of samples above LLOQ was observed in ‘Hawaii’, with 63% in summer-harvested leaves compared to 3% in winter-harvested leaves (**Table 5**). Almost all ‘Hawaii’ leaf samples with the harvested in the summer and dried at 25°C had above LLOQ levels of 7-hydroxymitragynine. Under 40 and 60°C, the number of samples with the above LLOQ level increased with the increase of withering duration. 7-hydroxymitragynine was above LLOQ in all samples after withering for 72 hours, regardless of the subsequent drying temperature. In contrast, for leaves harvested in the winter, the 7-hydroxymitragynine concentration in most of the ‘Hawaii’ leaf samples was below LLOQ. Additionally, no significant differences were noted among treatments in the average 7-hydroxymitragynine concentration. Interestingly, in ‘MR-Malaysian’, 7-hydroxymitragynine concentration was above LLOQ in almost all winter-harvested leaf samples, while they were all below LLOQ in summer-harvested samples, regardless of postharvest treatments (**Table 4**). The 7-hydroxymitragynine concentrations in ‘MR-Malaysian’ ranged from 0.023 to 0.035% in leaf extract, which were not significantly affected by the withering duration.

**Table 5:** Percentage of samples (%) with 7-hydroxymitragynine above LLOQ and 7-hydroxymitragynine content (% w/w ± _SE_) in leaf alkaloid extracts from Study I (cultivar Hawaii) under postharvest treatments by season.

		Withering duration	— 40°C	25°C	40°C	60°C	80°C
% Samples Above LLOQ	Summer	0 hour	NA	100	0	0	67
		12 hours		67	0	33	0
		24 hours		100	67	33	0
		72 hours		100	100	100	100
	Winter	0 hour	0	33	0	0	0
		12 hours	0	0	0	0	0
		24 hours	0	0	0	0	0
		72 hours	0	0	0	0	33
7-hydroxymitragynine content (%w/w)	Summer	0 hour	NA	0.013 ± 0.001	Below LLOQ	Below LLOQ	0.039 ± 0.002
		12 hours		0.020 ± 0.001	Below LLOQ	Below LLOQ	Below LLOQ
		24 hours		0.020 ± 0.001	0.016 ± 0.006	Below LLOQ	Below LLOQ
		72 hours		0.020 ± 0.001	0.018 ± 0.001	0.021 ± 0.001	0.017 ± 0.003
	Winter	0 hour	Below LLOQ	Below LLOQ	Below LLOQ	Below LLOQ	Below LLOQ
		12 hours	Below LLOQ	Below LLOQ	Below LLOQ	Below LLOQ	Below LLOQ
		24 hours	Below LLOQ	Below LLOQ	Below LLOQ	Below LLOQ	Below LLOQ
		72 hours	Below LLOQ	Below LLOQ	Below LLOQ	Below LLOQ	Below LLOQ

Data for alkaloid content were only reported with more than 2 valid data points. NA, not analyzed. LLOQ, Lower Limit of Quantification (limit of quantification = 0.005% in leaf alkaloid extract).

The withering duration also impacted the content of speciogynine, paynantheine, and speciociliatine in ‘Hawaii’ leaves. However, this effect was primarily observed at lower drying temperatures, with minimal variation occurring at drying temperatures above 40°C (**Table 6**). For speciogynine, a 12-hour withering period followed by drying at -40 and 25°C significantly increased its concentration by 48% and 37%, respectively. A 24-hour withering duration enhanced speciogynine concentration at 25°C, but not at other temperatures. Similarly, paynantheine concentration increased by 35-67% with a 12-hour withering period followed by drying at temperatures below 40°C, but the effect of withering duration was not observed at higher drying temperatures. For speciociliatine, under low drying temperatures (≤ 25°C), its concentration initially decreased as withering duration increased from 0 to 24 hours, then subsequently increased when withering duration was extended to 72 hours. However, this trend was not apparent at higher drying temperatures (> 40°C). Notably, a 12-hour withering period significantly reduced speciociliatine concentration by 0.36-fold at a drying temperature of 25°C, but this effect diminished once the withering duration exceeded 24 hours.

**Table 6:** Average speciogynine, paynantheine, corynantheidine, and speciociliatine content (% w/w ± _SE_) in leaf alkaloid extracts from Study I (cultivar Hawaii) under postharvest treatments.

Alkaloid name	Withering duration	— 40°C	25°C	40°C	60°C	80°C
Speciogynine	0 hour	0.65 ± 0.07 **bAB**	0.59 ± 0.06 **bB**	0.74 ± 0.06 **AB**	0.60 ± 0.06 **B**	0.79 ± 0.04 **A**
	12 hours	0.96 ± 0.06 **aA**	0.81 ± 0.03 **aAB**	0.72 ± 0.07 **B**	0.73 ± 0.03 **B**	0.73 ± 0.06 **B**
	24 hours	0.64 ± 0.04 **b**	0.80 ± 0.05 **a**	0.77 ± 0.04	0.61 ± 0.03	0.65 ± 0.03
	72 hours	0.73 ± 9.05 **ab**	0.72 ± 0.02 **ab**	0.63 ± 0.06	0.62 ± 0.02	0.67 ± 0.06
Paynantheine	0 hour	0.73 ± 0.07 **b**	0.79 ± 0.06 **b**	0.94 ± 0.09	0.8 ± 0.10	0.94 ± 0.05
	12 hours	1.22 ± 0.06 **a**	1.07 ± 0.02 **a**	0.95 ± 0.11	0.94 ± 0.04	0.95 ± 0.10
	24 hours	0.74 ± 0.04 **b**	1.00 ± 0.09 **ab**	0.98 ± 0.08	0.82 ± 0.07	0.82 ± 0.06
	72 hours	0.88 ± 0.06 **ab**	0.97 ± 0.04 **ab**	0.84 ± 0.07	0.84 ± 0.05	0.91 ± 0.10
Corynantheidine	0 hour	0.10 ± 0.01	0.08 ± 0.01	0.06 ± 0.01	0.05 ± 0.001	0.09 ± 0.01
	12 hours	0.08 ± 0.01	0.08 ± 0.01	0.10 ± 0.02	0.09 ± 0.02	0.07 ± 0.01
	24 hours	0.09 ± 0.03	0.10 ± 0.01	0.06 ± 0.01	0.08 ± 0.01	0.08 ± 0.01
	72 hours	0.10 ± 0.02	0.11 ± 0.01	0.09 ± 0.02	0.06 ± 0.002	0.09 ± 0.02
Speciociliatine	0 hour	0.99 ± 0.14 **abA**	0.78 ± 0.19 **abAB**	0.55 ± 0.16 **AB**	0.42 ± 0.14 **AB**	0.32 ± 0.03 **B**
	12 hours	0.45 ± 0.09 **ab**	0.28 ± 0.04 **c**	0.37 ± 0.15	0.34 ± 0.07	0.35 ± 0.10
	24 hours	0.34 ± 0.19 **b**	0.38 ± 0.12 **bc**	0.20 ± 0.06	0.50 ± 0.18	0.29 ± 0.11
	72 hours	1.02 ± 0.17 **aA**	0.91 ± 0.12 **aA**	0.47 ± 0.13 **AB**	0.32 ± 0.09 **B**	0.67 ± 0.24 **AB**

Mean comparisons were conducted within each alkaloid using data pooled from two seasons. Lowercase letters indicate mean separation for withering duration; uppercase letters indicate mean separation for drying temperature. Means sharing different letters are statistically different by Tukey’s HSD test at P < 0.05.

In contrast, a 12-hour withering period only significantly increased speciogynine concentration in ‘MR-Malaysian’ by 22% under a high drying temperature (60°C) in the winter-harvested samples (**Table 4**). Moreover, the concentrations of mitraciliatine and isopaynantheine increased by 61% and 59%, respectively, under 25°C in winter-harvested leaves, whereas the concentration of corynoxine A was reduced by 23%.

Additionally, withering duration did not have a noticeable effect on corynantheidine concentration in both cultivars, as well as on paynantheine, speciociliatine and corynoxine B concentration in ‘MR-Malaysian’ (**Tables 4**, **6**). Furthermore, corynoxine A and B concentrations were below LLOQ in ‘Hawaii’. No isospeciofoline, mitraphylline, or ajmalicine were above LLOQ in either cultivar (data not shown).

### 3.3 Effects of drying temperature on bioactive compounds

Overall, low drying temperatures preserved mitragynine in kratom leaf extracts better than high drying temperatures. For example, the overall mitragynine concentration in leaves dried at 25°C was 3-26% higher than that dried at 60°C (**Figure 2**). The drying temperature had a more significant effect when leaves experienced no withering duration, and this influence diminished once the withering duration exceeded 24 hours (**Tables 3**, **4**).

The greatest number of samples with 7-hydroxymitragynine above LLOQ in kratom ‘Hawaii’ was observed at a drying temperature of 25°C compared to other drying temperatures (**Table 5**). In contrast, drying temperatures had no significant effect on the number of samples with 7-hydroxymitragynine above LLOQ in ‘MR-Malaysian’ (**Table 4**). Additionally, in ‘Hawaii’, there were a few differences between the drying temperature treatments (**Table 5**). Conversely, in ‘MR-Malaysian’, 7-hydroxymitragynine concentration was 21-39% higher when dried at 25°C compared to drying at 60°C (**Table 4**).

For speciogynine, a drying temperature of 80°C resulted in the highest concentration when leaves were dried without withering in kratom ‘Hawaii’ (**Table 6**). In contrast, under a 12-hour withering period, drying at lower temperatures resulted in higher speciogynine concentration. Similarly, for paynantheine, although not significant, ‘Hawaii’ had the highest concentration under high drying temperatures when leaves experienced no withering. However, with a 12-hour withering period, leaves achieved the highest paynantheine concentration under low drying temperatures. Conversely, speciociliatine had the highest concentration under freeze-drying without withering, with its concentration being threefold higher compared to leaves dried at 80°C. Under no withering conditions, speciociliatine concentration generally decreased in ‘Hawaii’ as the drying temperature increased. However, in ‘MR-Malaysian’, its concentration typically increased, albeit not significantly, with the increase in drying temperature (**Tables 4**, **6**).

Drying temperatures had little effect on corynantheidine in kratom ‘Hawaii’, whereas higher drying temperatures generally decreased its concentration in ‘MR-Malaysian’ (**Tables 4**, **6**). Interestingly, in kratom ‘MR-Malaysian’, a high drying temperature at 60 °C significantly increased the accumulation of some oxindole alkaloids. For example, the concentration of corynoxine B increased by 32-124% in winter-harvested leaves, regardless of withering durations (**Table 4**). Additionally, when leaves were dried at 25°C, corynoxine A and 9-hydroxycorynantheidine were above the LLOQ in 100% and 90% of the samples, respectively. However, both compounds fell below the LLOQ in samples dried at 60°C. Furthermore, drying temperatures had little effect on mitraciliatine, isopaynantheine, and corynoxine A in kratom ‘MR-Malaysian’ (**Table 4**).

## 4 Discussion

Kratom products sold in Western markets are often categorized into different strains, commonly labeled as red, green, and white. These classifications are most commonly based on the color of the powdered product rather than the actual leaf vein color and variety. Marketing narratives and anecdotal reports from kratom users frequently suggest that different strains produce distinct physiological effects. For instance, red kratom strains are often associated with anxiolytic and calming effects, while green strains are marketed as being more energizing, stimulating, and mood-enhancing (Huisman et al., 2023). Our study demonstrates that postharvest processing conditions significantly influence the final color of kratom powder, even when derived from the same batch of leaves. More importantly, our findings suggest that the powder color does not necessarily correlate with alkaloid composition, but rather serves as an indicator of the postharvest environment. For example, in kratom ‘Hawaii’, when leaves were dried immediately without a withering period, the key alkaloid contents, including mitragynine, speciogynine, and paynantheine, were similar between leaves dried at 25°C and 60°C. However, the visual appearance of the powder differed significantly, with samples dried at 60°C exhibiting a reddish hue, whereas those dried at 25°C retained a green color. These findings align with the survey results reported by Huisman et al. (2023), who found no significant differences in alkaloid profiles among six commercial kratom products labeled with different strains. Additionally, our study revealed that even when similar in color, kratom powder can present different alkaloid profiles. For example, when kratom leaves were dried at 25°C, both the 0-hour and 12-hour withering treatments produced green-colored powders. However, the key alkaloid concentrations, particularly mitragynine, speciogynine, and paynantheine, were significantly higher in leaves that underwent a 12-hour withering process compared to those with no withering. It is worth noting that although Huisman et al. (2023) did not observe any significant differences in alkaloid levels between strains, users often report distinct subjective experiences associated with different strains. These perceptions may be influenced by marketing strategies and user expectations (Huisman et al., 2023), or possibly by non-alkaloid constituents not assessed in this study. Collectively, these findings suggest that marketing kratom strains based on powder color can be misleading and may not accurately reflect product quality or chemical composition.

There were notable and at times opposing differences in alkaloid content in two kratom cultivars, Hawaii and MR-Malaysian, in response to environmental changes, which highlights the potential influence of genotype and seasonal variation on alkaloid biosynthesis. Withering duration significantly influenced mitragynine concentration in ‘Hawaii’, whereas it had a minimal effect on ‘MR-Malaysian’. In contrast, drying temperature had a greater effect on ‘MR-Malaysian’ than on ‘Hawaii’. As outlined in the Methods section, two cultivars were studied in both summer and winter, allowing for a comparison across seasonal conditions. Mitragynine concentrations in ‘Hawaii’ were generally higher during summer, while concentrations in cultivar ‘MR-Malaysian’ tended to be higher during winter. A similar seasonal contrast was observed with 7-hydroxymitragynine, a key metabolite of mitragynine. In ‘Hawaii’, 7-hydroxymitragynine was detectable only during the summer, whereas in ‘MR-Malaysian’, it was detectable only in the winter. Additionally, the alkaloids corynoxine A and B were consistently detected in ‘MR-Malaysian’ but were undetectable in ‘Hawaii’ regardless of seasons. This suggests that potential genetic differences occur in the biosynthesis of bioactive alkaloid compounds. Furthermore, greater variability in alkaloid concentration was observed in ‘MR-Malaysian’ between seasons compared to ‘Hawaii’, indicating that ‘MR-Malaysian’ may be more sensitive to subtle environmental or seasonal fluctuations. However, detailed environmental data were not collected for the mother stock’s growing conditions across seasons in this study, limiting the ability to perform a more in-depth analysis of environmental influences during this period. Interestingly, Chear et al. (2021) also reported substantial variability in alkaloid profiles among naturally growing wild kratom trees in Malaysia, suggesting the presence of genetic differences within wild populations. This raises the possibility that kratom beverages consumed in Southeast Asia may often be derived from a mixture of genetically diverse leaves. Collectively, these results provide evidence that cultivar genetics may play a significant role in influencing the abundance and the seasonal dynamics of key alkaloids in kratom. Further research is warranted to investigate how specific environmental variables influence alkaloid expression in genetically distinct kratom cultivars.

The withering process, primarily driven by gradual water loss, imposes external stress on plant tissues, which can activate a cascade of physiological and biochemical signaling pathways. This stress response often leads to the enhanced biosynthesis of various secondary metabolites, including alkaloids, as part of the plant’s adaptive defense mechanisms (Dai et al., 2025). Significant changes in primary metabolism also occur. Macromolecules, such as proteins, may undergo degradation, providing precursors and energy needed for the production of secondary metabolites. For instance, approximately 15% of chlorophyll content has been reported to degrade during withering (Sanyal, 2011). Additionally, moisture removal caused by withering can concentrate the cell sap, increasing enzyme activity and forming high molecular units from low molecular units (Deb and Jolvis Pou, 2016). These biochemical transformations create a favorable environment for the activation of specialized metabolism.

**Figure 3:**
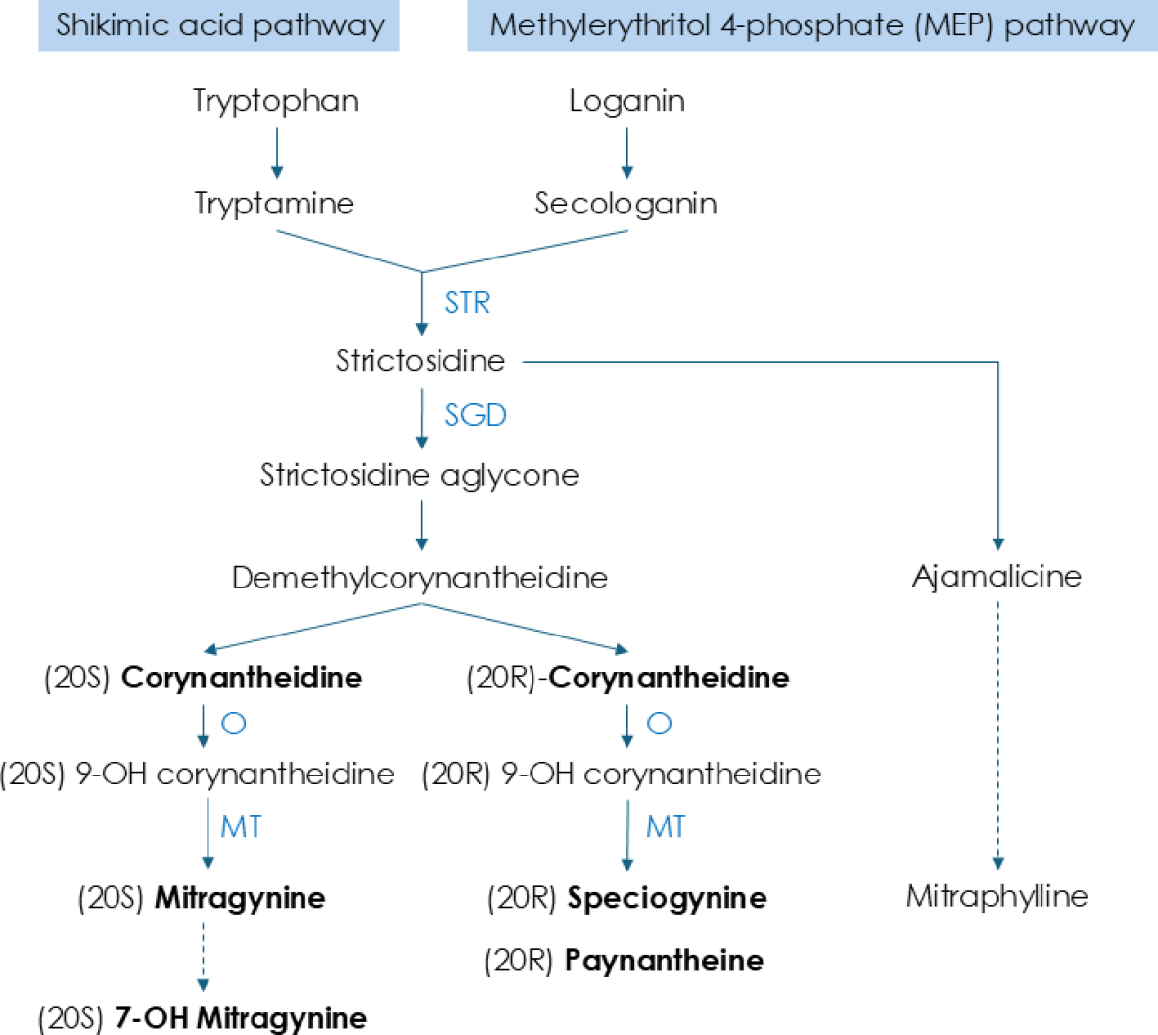
Biosynthesis pathways for key alkaloids in kratom. Summarized and simplified from Lopes et al., 2019; Kim et al., 2023; Schotte et al., 2023.

In the present study, we observed that the availability of key alkaloids, including mitragynine, speciogynine, paynantheine, and corynantheidine, generally increased with the increase of withering duration at ambient temperatures (25°C), particularly in kratom ‘Hawaii’. This trend is consistent with findings in tea leaves where caffeine levels significantly increase by the end of the withering phase, likely due to the conversion of its precursor, theobromine, and enhanced activity of tea caffeine synthase under low-temperature conditions (Xia et al., 2017; Chen et al., 2020; Yu et al., 2020; Qi et al., 2024). Drawing on a simplified model of the kratom indole alkaloid biosynthesis pathway compiled from the current literature (Lopes et al., 2019; Kim et al., 2023; Schotte et al., 2023) (**Figure 3**), we propose that withering enhanced the synthesis of substances upstream of strictosidine aglycone, such as free amino acids, to enhance the overall indole alkaloid biosynthesis pathways in kratom. This hypothesis is based on our results that a 12-hour withering period yielded the highest quality kratom product. Beyond this duration, levels of some key alkaloids may begin to decline, potentially due to the depletion of precursors or the activation of feedback inhibition mechanisms. Similarly, in tea leaves, there was an increase in free amino acids during withering, attributing to proteolysis and the upregulation of protein degradation enzymes and amino acid synthases (Qi et al., 2024). Furthermore, our finding aligns with observations in tea, where alkaloid-related metabolic activity is most pronounced during the initial 6 hours of withering and gradually diminishes beyond 18 hours (Dai et al., 2025). Accordingly, tea’s optimal chemical withering duration is approximately 14 hours and should not exceed 18 hours (Deb and Jolvis Pou, 2016; Owuor and Orchard, 1992; Omiadze et al., 2014). These collective findings indicate the critical role of withering duration in modulating alkaloid biosynthesis and provide insight into optimizing postharvest handling strategies for kratom.

It is important to note that in our study, the withering experiment was conducted using a single layer of kratom leaves, allowing for uniform exposure to ambient conditions. This methodology may differ from commercial practices, where leaves are typically stacked in layers during the withering process and require periodic manual turnovers to promote uniformity in drying. Several withering systems, including trough withering, solar withering, heated withering, indoor withering, and controllable light withering, have been developed over the years in the tea industry to optimize moisture removal and enhance the development of secondary metabolites and aroma (Qi et al., 2024). Similar systems are likely being adopted in the kratom industry in Southeast Asia to enhance product quality and consistency. Further research evaluating how different withering systems and stacking depths influence the biochemical dynamics of alkaloid biosynthesis in kratom is warranted.

In kratom ‘Hawaii’, several alkaloids, including mitragynine, 7-hydroxymitragynine, speciogynine, paynantheine, and corynantheidine, were found in the highest concentration in kratom powders dried at high drying temperatures without prior withering. However, when withering exceeded 12 hours, the greatest alkaloid concentrations were generally observed under lower drying temperatures. A similar trend was noted in kratom ‘MR-Malaysian’, where the highest alkaloid concentrations were typically achieved under lower drying temperatures. These patterns suggest that the biosynthesis of kratom alkaloids may be mediated by enzymes whose activities may be sensitive to both temperature and time. In a new equilibrium model of the enzyme thermal behavior proposed by Daniel and Danson (2010), enzymes exhibit higher optimal temperatures for activity at the onset of a biological process (time = 0), but this optimal temperature decreases as the process progresses. For instance, alkaline phosphatase activity peaked at approximately 315 K (41.9°C) at time zero but shifted to an optimum of 305–310 K (31.9–36.8°C) after 3500 seconds. A similar trend was observed in *Caldocellulosiruptor saccharolyticus* β-glucosidase, where peak activity was initially recorded at 350 to 360 K (76.9 to 86.9°C) but shifted to 330 K (56.9°C) after 250 seconds. This supports our hypothesis that the enzymes involved in mitragynine and other kratom indole alkaloid biosynthesis may be influenced by both withering duration and drying temperature. As time progresses during postharvest processing, a lower temperature environment may generally be favorable for enzyme activity and alkaloid accumulation.

Mitragynine and its metabolite, 7-hydroxymitragynine, are the key psychoactive alkaloids in kratom that have attracted significant interest due to their pharmacological activities. Mitragynine is the predominant alkaloid in kratom, known to interact with opioid, adrenergic, and serotonergic receptors, and produces a range of pharmacological effects, including both sedative and stimulant properties, as well as anti-inflammatory and antinociceptive actions (Annuar et al., 2024). In our study, mitragynine concentrations in kratom powders processed from harvested leaves generally ranged from 0.5-1.5% in cultivar Hawaii and 0.2-1.4% in cultivar MR-Malaysian. In corresponding alkaloid extracts, concentrations range from 4–8.5% in ‘Hawaii’ and 2–8% in ‘MR-Malaysian’, depending on postharvest treatments and harvest seasons. These values are comparable to the mitragynine content found in native kratom trees from Thailand (0.4-2.2% in leaves; Leksungnoen et al., 2022, 2025) and Malaysia (0.9-1.8% in leaves (Chear et al., 2021), with differences likely attributed to environmental conditions, plant age, and genetic differences among cultivars.

In contrast, 7-hydroxymitragynine is a potent μ-opioid receptor agonist associated with strong analgesic activity but also with potential risks such as respiratory depression at higher doses, raising concerns regarding its abuse liability (Hemby et al., 2019; McCurdy et al., 2024). Although research remains limited, varying concentrations of 7-hydroxymitragynine have been reported in both kratom leaf samples and commercial kratom products. In commercial kratom products available in the US market (i.e., powders or crumbs from dried leaves), 7-hydroxymitragynine typically ranges from 0.01% to 0.21% (Sharma et al., 2025). Among native trees, 7-hydroxymitragynine concentrations have been reported to range from 0.003% to 0.012% in Thai kratom leaves and from 0.03% to 0.15% in dried leaves from Malaysian trees (Leksungnoen et al., 2022; Kikura-Hanajiri et al., 2009; Chear et al., 2021). In our previous studies, 7-hydroxymitragynine was not detected in kratom leaf samples, likely due to the use of a different cultivar that also produced low levels of mitragynine (Zhang et al., 2020, 2022). In the current study, the concentration of 7-hydroxymitragynine in the leaf alkaloid extract was 0.01–0.04% for ‘Hawaii’ and 0.02–0.04% for ‘MR-Malaysian’, corresponding to 0.002–0.004% and 0.003–0.006%, respectively, in the leaf material. Despite analyzing concentrated extracts, the calculated 7-hydroxymitragynine content in the dry leaves are 2- to 5-fold lower than the lowest 7-hydroxymitragynine concentrations reported in commercial kratom leaf products (Sharma et al., 2025) and more than 10-fold lower than those found in native Malaysian trees (Chear et al., 2021). Additionally, the highest 7-hydroxymitragynine concentration found in this study is unlikely to be clinically significant, especially considering that 7-hydroxymitragynine has limited dissolution when consumed through leaf material and exhibits low oral bioavailability following intragastric administration (Chiang et al., 2025). Collectively, these findings suggest that the germplasm we selected can achieve mitragynine levels comparable to those of Southeast Asian varieties when cultivated using appropriate techniques, such as optimized postharvest processing and seasonal harvesting, while maintaining significantly lower concentrations of 7-hydroxymitragynine, which reduces the potential for drug abuse. These results further reiterate that kratom should be consumed in its traditional, unadulterated form, such as fresh or dried leaf material, which naturally contains low amounts of 7-hydroxymitragynine, rather than in adulterated forms, such as concentrated extracts, that carry a higher risk of misuse (Grundmann et al., 2024).

The synthesis of 7-hydroxymitragynine remains unclear, and questions persist regarding whether it is naturally produced by the plant or formed during postharvest processing through oxidative transformations (Ganasan et al., 2024). Some studies have reported the presence of 7-hydroxymitragynine in “fresh leaves” (Chear et al., 2021; Karunakaran et al., 2024), however, these leaves were not preserved in liquid nitrogen immediately after harvest. As a result, ongoing biological and enzymatic activities may have influenced alkaloid biosynthesis prior to analysis. Therefore, further research using leaves preserved immediately after harvest is warranted to determine whether 7-hydroxymitragynine is synthesized by the plant itself or not.

Several studies support the hypothesis that 7-hydroxymitragynine is a postharvest oxidative or metabolic derivative of mitragynine, formed through processes such as oxidation or hydrolysis that may be influenced by environmental factors, including temperature and humidity, during storage and handling (Chear et al., 2021; Ganasan et al., 2024; Karunakaran et al., 2024). However, this does not fully explain our observation that 7-hydroxymitragynine was above LLOQ only during the summer months in cultivar Hawaii and only during the winter months in cultivar MR-Malaysian, regardless of postharvest treatments. Additionally, Kikura-Hanajiri et al. (2009) reported that kratom trees with low mitragynine production also had significantly lower levels of 7-hydroxymitragynine compared to those with high mitragynine production. This finding aligns with our previous results, where 7-hydroxymitragynine was below LLOQ in kratom trees that produced low levels of mitragynine (Zhang et al., 2020, 2022). Together, these findings suggest that kratom trees producing higher levels of mitragynine also tend to produce more 7-hydroxymitragynine. Collectively, we believe that the presence and concentration of 7-hydroxymitragynine in kratom products could be influenced by both endogenous biosynthesis within the plant, although no biochemical evidence of the responsible enzymes has been identified to date, and by the postharvest conversion of mitragynine. Genetic factors, potentially modulated by seasonal variation, likely play a more prominent role in determining its accumulation.

## 5 Conclusion

Kratom alkaloid biosynthesis was influenced by postharvest handling, genotype, and seasonal variation. Leaves withered for 12 hours and dried at low temperatures retained the highest levels of mitragynine and generally yielded greater concentrations of desirable alkaloids. Our findings challenge a common marketing misconception, demonstrating that the color of kratom powder is determined by postharvest processing conditions rather than alkaloid composition. The two cultivars examined in this study produced mitragynine levels comparable to those reported from trees grown in Southeast Asia but exhibited significantly lower levels of 7-hydroxymitragynine. Overall, the presence and concentration of 7-hydroxymitragynine in kratom products appear to result from a combination of endogenous biosynthesis and postharvest transformation.

## Data Availability

The raw data supporting the conclusions of this article will be made available by the authors, without undue reservation.

## References

[B1] Ademiluyi A. O. Aladeselu O. H. Oboh G. Boligon A. A. (2018). Drying alters the phenolic constituents, antioxidant properties, α-amylase, and α-glucosidase inhibitory properties of *Moringa* (*Moringa oleifera*) leaf. Food Sci. Nutr. 6, 2123–2133. doi: 10.1002/fsn3.770, PMID: 30510713 PMC6261129

[B2] Annuar N. A. K. Azlan U. K. Mediani A. Tong X. Han R. Al-Olayan E.. (2024). An insight review on the neuropharmacological effects, mechanisms of action, pharmacokinetics and toxicity of mitragynine. Biomedicine Pharmacotherapy 171, 116134. doi: 10.1016/j.biopha.2024.116134, PMID: 38219389

[B3] Chear N. J.-Y. León F. Sharma A. Kanumuri S. R. R. Zwolinski G. Abboud K. A.. (2021). Exploring the chemistry of alkaloids from Malaysian *Mitragyna* sp*eciosa* (Kratom) and the role of oxindoles on human opioid receptors. J. Natural Products 84, 1034–1043. doi: 10.1021/acs.jnatprod.0c01055, PMID: 33635670 PMC8693998

[B4] Chen Q. Shi J. Mu B. Chen Z. Dai W. Lin Z. (2020). Metabolomics combined with proteomics provides a novel interpretation of the changes in nonvolatile compounds during white tea processing. Food Chem. 332, 127412. doi: 10.1016/j.foodchem.2020.127412, PMID: 32623128

[B5] Chiang Y. H. Kanumuri S. R. R. Kuntz M. A. Senetra A. S. Berthold E. C. Kamble S. H.. (2025). *In vitro* and *in vivo* pharmacokinetic characterization of 7-hydroxymitragynine, an active metabolite of mitragynine, in sprague-dawley rats. Eur. J. Drug Metab. Pharmacokinet. 50, 205–218. doi: 10.1007/s13318-025-00939-2, PMID: 40119246

[B6] Cinosi E. Martinotti G. Simonato P. Singh D. Demetrovics Z. Roman-Urrestarazu A.. (2015). Following “the roots” of kratom (*Mitragyna* sp*eciosa*): The evolution of an enhancer from a traditional use to increase work and productivity in Southeast Asia to a recreational psychoactive drug in western countries. BioMed. Res. Int. 2015, 968786. doi: 10.1155/2015/968786, PMID: 26640804 PMC4657101

[B7] Dai Y. Yang T. Luo J. Fang S. Zhang T. Li Q.. (2025). Changes in alkaloids and their related metabolites during the processing of ‘Qiancha 1′ white tea based on transcriptomic and metabolomic analysis. LWT 218, 117435. doi: 10.1016/j.lwt.2025.117435

[B8] Daniel R. M. Danson M. J. (2010). A new understanding of how temperature affects the catalytic activity of enzymes. Trends Biochem. Sci. 35, 584–591. doi: 10.1016/j.tibs.2010.05.001, PMID: 20554446

[B9] Deb S. Jolvis Pou K. R. (2016). A review of withering in the processing of black tea. J. Biosyst. Eng. 41, 365–372. doi: 10.5307/JBE.2016.41.4.365

[B10] Ganasan J. Karunakaran T. Marimuthu Y. Rusmadi N. N. Firouz N. S. Jenis J.. (2024). Chemistry and toxicity of 7-hydroxymitragynine (7-OHMG): An updated review on the oxidized derivative of mitragynine. Phytochem. Rev., 1–14. doi: 10.1007/s11101-024-10029-x

[B11] Grundmann O. (2017). Patterns of kratom use and health impact in the US—results from an online survey. Drug Alcohol Depend. 176, 63–70. doi: 10.1016/j.drugalcdep.2017.03.007, PMID: 28521200

[B12] Grundmann O. Garcia-Romeu A. McCurdy C. R. Sharma A. Smith K. E. Swogger M. T.. (2024). Not all kratom is equal: the important distinction between native leaf and extract products. Addiction 119, 202–203. doi: 10.1111/add.16366, PMID: 37814405

[B13] Grundmann O. Hendrickson R. G. Greenberg M. I. (2023). Kratom: History, pharmacology, current user trends, adverse health effects and potential benefits. Disease-a-Month 69, 101442. doi: 10.1016/j.disamonth.2022.101442, PMID: 35732553

[B14] Hassan Z. Muzaimi M. Navaratnam V. Yusoff N. H. Suhaimi F. W. Vadivelu R.. (2013). From kratom to mitragynine and its derivatives: Physiological and behavioural effects related to use, abuse, and addiction. Neurosci. Biobehav. Rev. 37, 138–151. doi: 10.1016/j.neubiorev.2012.11.012, PMID: 23206666

[B15] Hemby S. E. McIntosh S. Leon F. Cutler S. J. McCurdy C. R. (2019). Abuse liability and therapeutic potential of the *Mitragyna* sp*eciosa* (kratom) alkaloids mitragynine and 7-hydroxymitragynine. Addict. Biol. 24, 874–885. doi: 10.1111/adb.12639, PMID: 29949228

[B16] Horie S. Koyama F. Takayama H. Ishikawa H. Aimi N. Ponglux D.. (2005). Indole alkaloids of a Thai medicinal herb, *Mitragyna* sp*eciosa*, that has an opioid agonistic effect in Guinea-pig ileum. Planta Med. 71, 231–236. doi: 10.1055/s-2005-837822, PMID: 15770543

[B17] Huisman G. Menke M. Grundmann O. Schreiber R. Mason N. (2023). Examining the psychoactive differences between kratom strains. Int. J. Environ. Res. Public Health 20, 6425. doi: 10.3390/ijerph20146425, PMID: 37510657 PMC10379209

[B18] Jia X. Zhang Q. Chen M. Wang Y. Lin S. Pan Y.. (2023). Analysis of the effect of different withering methods on tea quality based on transcriptomics and metabolomics. Front. Plant Sci. 14. doi: 10.3389/fpls.2023.1235687, PMID: 37780509 PMC10538532

[B19] Kamble S. H. Berthold E. C. King T. I. Raju Kanumuri S. R. Popa R. Herting J. R.. (2021). Pharmacokinetics of eleven kratom alkaloids following an oral dose of either traditional or commercial kratom products in rats. J. Natural Products 84, 1104–1112. doi: 10.1021/acs.jnatprod.0c01163, PMID: 33620222 PMC8694001

[B20] Karunakaran T. Vicknasingam B. Chawarski M. C. (2024). Phytochemical analysis of water and ethanol liquid extracts prepared using freshly harvested leaves of *Mitragyna* sp*eciosa* (Korth.). Natural Product Res. 39 (15), 4480–4487. doi: 10.1080/14786419.2024.2362428, PMID: 38842220

[B21] Kikura-Hanajiri R. Kawamura M. Maruyama T. Kitajima M. Takayama H. Goda Y. (2009). Simultaneous analysis of mitragynine, 7-hydroxymitragynine, and other alkaloids in the psychotropic plant “kratom” (*Mitragyna* sp*eciosa*) by LC-ESI-MS. Forensic Toxicol. 27, 67–74. doi: 10.1007/s11419-009-0070-5

[B22] Kim K. Shahsavarani M. Garza-García J. J. O. Carlisle J. E. Guo J. De Luca V.. (2023). Biosynthesis of kratom opioids. New Phytol. 240, 757–769. doi: 10.1111/nph.19162, PMID: 37518950

[B23] Kruegel A. C. Gassaway M. M. Kapoor A. Váradi A. Majumdar S. Filizola M.. (2016). Synthetic and receptor signaling explorations of the mitragyna alkaloids: Mitragynine as an atypical molecular framework for opioid receptor modulators. J. Am. Chem. Soc. 138, 6754–6764. doi: 10.1021/jacs.6b00360, PMID: 27192616 PMC5189718

[B24] Leksungnoen N. Andriyas T. Ku-Or Y. Chongdi S. Tansawat R. Aramrak A.. (2025). The effect of light intensity and polyethylene-glycol-induced water stress on the growth, mitragynine accumulation, and total alkaloid content of kratom (*Mitragyna* sp*eciosa*). Horticulturae 11, 272. doi: 10.3390/horticulturae11030272

[B25] Leksungnoen N. Andriyas T. Ngernsaengsaruay C. Uthairatsamee S. Racharak P. Sonjaroon W.. (2022). Variations in mitragynine content in the naturally growing kratom (*Mitragyna* sp*eciosa*) population of Thailand. Front. Plant Sci. 13. doi: 10.3389/fpls.2022.1028547, PMID: 36388525 PMC9648690

[B26] León F. Obeng S. Mottinelli M. Chen Y. King T. I. Berthold E. C.. (2021). Activity of *Mitragyna* sp*eciosa* (“kratom”) alkaloids at serotonin receptors. J. Medicinal Chem. 64, 13510–13523. doi: 10.1021/acs.jmedchem.1c00726, PMID: 34467758 PMC9235362

[B27] Lopes A. A. Chioca B. Musquiari B. Crevelin E. J. Franca S. D. C. Fernandes da Silva M. F. D. G.. (2019). Unnatural spirocyclic oxindole alkaloids biosynthesis in *Uncaria guianensis* . Sci. Rep. 9, 1–8. doi: 10.1038/s41598-019-47706-3, PMID: 31383908 PMC6683290

[B28] Manzano Santana P. Quijano-Avilés M. Chóez-Guaranda I. Barragán Lucas A. Viteri Espinoza R. Martínez D.. (2018). Effect of drying methods on physical and chemical properties of *Ilex guayusa* leaves. Rev. Facultad Nacional Agronomía Medellín 71, 8617–8622. doi: 10.15446/rfnam.v71n3.71667

[B29] McCurdy C. R. Sharma A. Smith K. E. Veltri C. A. Weiss S. T. White C. M.. (2024). An update on the clinical pharmacology of kratom: Uses, abuse potential, and future considerations. Expert Rev. Clin. Pharmacol. 17, 131–142. doi: 10.1080/17512433.2024.2305798, PMID: 38217374 PMC10846393

[B30] Obeng S. Kamble S. H. Reeves M. E. Restrepo L. F. Patel A. Behnke M.. (2019). Investigation of the adrenergic and opioid binding affinities, metabolic stability, plasma protein binding properties, and functional effects of selected indole-based kratom alkaloids. J. Medicinal Chem. 63, 433–439. doi: 10.1021/acs.jmedchem.9b01465, PMID: 31834797 PMC7676998

[B31] Omiadze N. T. Mchedlishvili N. I. Rodrigez-López J. N. Abutidze M. O. Sadunishvili T. A. Pruidze N. G. (2014). Biochemical processes at the stage of withering during black tea production. Appl. Biochem. Microbiol. 50, 394–397. doi: 10.1134/S0003683814040105, PMID: 25707121

[B32] Owuor P. O. Orchard J. E. (1992). Effects of storage time in a two-stage withering process on the quality of seedling black tea. Food Chem. 45, 45–49. doi: 10.1016/0308-8146(92)90040-B

[B33] Palamar J. J. (2021). Past-year kratom use in the US: estimates from a nationally representative sample. Am. J. Prev. Med. 61, 240–245. doi: 10.1016/j.amepre.2021.03.014, PMID: 34027890 PMC8319032

[B34] Qi D. Shi Y. Lu M. Ma C. Dong C. (2024). Effect of withering/spreading on the physical and chemical properties of tea: A review. Compr. Rev. Food Sci. Food Saf. 23, e70010. doi: 10.1111/1541-4337.13010, PMID: 39267185

[B35] Sanyal S. (2011). “Tea manufacturing manual,” in Tea research association, tocklai experimental station(Jorhat, Tocklai Tea Research Institute, Jorhat, India).

[B36] Schotte C. Jiang Y. Grzech D. Dang T. T. T. Laforest L. C. León F.. (2023). Directed biosynthesis of mitragynine stereoisomers. J. Am. Chem. Soc 145, 4957–4963. doi: 10.1021/jacs.2c13198, PMID: 36883326 PMC9999412

[B37] Sharma A. Kamble S. H. León F. Chear N. J. Y. King T. I. Berthold E. C.. (2019). Simultaneous quantification of ten key kratom alkaloids in Mitragyna speciosa leaf extracts and commercial products by ultra-performance liquid chromatography-tandem mass spectrometry. Drug Test. Anal. 11, 1162–1171. doi: 10.1002/dta.2604, PMID: 30997725 PMC7927418

[B38] Sharma A. Smith K. E. Kuntz M. A. Berthold E. C. Elashkar O. I. Guadagnoli N.. (2025). Chemical analysis and alkaloid intake for kratom products available in the United States. Drug Testing Anal., 1–11. doi: 10.1002/dta.3906, PMID: 40377101

[B39] Suwanlert S. (1975). A study of kratom eaters in Thailand. Bull. Narc. 27, 21–27. Available online at: https://www.unodc.org/unodc/en/data-and-analysis/bulletin/bulletin_1975-01-01_3_page003.html (Accessed May 20, 2025)., PMID: 1041694

[B40] Syarma R. Kartikawati S. M. Setyawati D. (2019). Characteristics and knowledge of the people of Entibab village about the use of the kratom (Mitragyna speciosa) plant in the Kapuas Hulu District. J. Hutan Lestari 11, 75–87. doi: 10.26418/jhl.v11i1.60416

[B41] Váradi A. Marrone G. F. Palmer T. C. Narayan A. Szabó M. R. Le Rouzic V.. (2016). Mitragynine/corynantheidine pseudoindoxyls as opioid analgesics with mu agonism and delta antagonism, which do not recruit β-arrestin-2. J. Med. Chem. 59, 8381–8397. doi: 10.1021/acs.jmedchem.6b00748, PMID: 27556704 PMC5344672

[B42] Xia E.-H. Zhang H.-B. Sheng J. Li K. Zhang Q.-J. Kim C.. (2017). The tea tree genome provides insights into tea flavor and independent evolution of caffeine biosynthesis. Mol. Plant 10, 866–877. doi: 10.1016/j.molp.2017.04.002, PMID: 28473262

[B43] Ye Y. Yan J. Cui J. Mao S. Li M. Liao X.. (2018). Dynamic changes in amino acids, catechins, caffeine and gallic acid in green tea during withering. J. Food Compos. Anal. 66, 98–108. doi: 10.1016/j.jfca.2017.12.003

[B44] Yu X. Li Y. He C. Zhou J. Chen Y. Yu Z.. (2020). Nonvolatile metabolism in postharvest tea (*Camellia sinensis* L.) leaves: Effects of different withering treatments on nonvolatile metabolites, gene expression levels, and enzyme activity. Food Chem. 327, 126992. doi: 10.1016/j.foodchem.2020.126992, PMID: 32447133

[B45] Zhang M. Sharma A. León F. Avery B. Kjelgren R. McCurdy C. R.. (2020). Effects of nutrient fertility on growth and alkaloidal content in *Mitragyna* sp*eciosa* (Kratom). Front. Plant Sci. 11. doi: 10.3389/fpls.2020.597696, PMID: 33408731 PMC7779599

[B46] Zhang M. Sharma A. León F. Avery B. Kjelgren R. McCurdy C. R.. (2022). Plant growth and phytoactive alkaloid synthesis in kratom [*Mitragyna* sp*eciosa* (Korth.)] in response to varying radiance. PloS One 17, e0259326. doi: 10.1371/journal.pone.0259326, PMID: 35472200 PMC9041851

[B47] Zuiderveen G. H. Burkhart E. P. Lambert J. D. (2021). Influence of postharvest drying temperatures on alkaloid levels in goldenseal (*Hydrastis canadensis* L.). HortScience 56, 242–243. doi: 10.21273/HORTSCI15689-20

