# Supplementary material for: Alkaloid biosynthesis in medicinal crop kratom (Mitragyna speciosa) varies with postharvest, genetic, and seasonal factors
Source: Front Plant Sci. 2025 Sep 29;16:1653916. doi: 10.3389/fpls.2025.1653916 (PMC12516786; doi:10.3389/fpls.2025.1653916)
Supplement: Supplementary file 1 [file DataSheet1.docx]

# Supplementary Information

Below details the maintenance and management of kratom mother stocks in two studies.

*Study I*

Kratom ‘Hawaii’ mother stocks were cultivated in a corrugated, opaque polycarbonate greenhouse located in Apopka, Florida, United States (28.64 N, 81.55 W) under natural daylight. Trees were maintained in 76 L containers filled with soilless substrate containing 50% peat (Florida: Canadian peat = 1:1), 30% pine bark, and 20% perlite, and supplied with top-dressed control-release fertilizer (Osmocote 15-9-12; Everris NA Inc., Dublin, OH, United States) as needed. Auto irrigation was applied daily using a micro-irrigation spray stake (Brown Mini Flow Spot-Spitter, Primerus Products, LLC., Encinitas, CA, United States) controlled by a programmable irrigation controller (Sterling 12; Superior Controls Co., Inc., Valencia, CA).

*Study II*

‘MR-Malaysian’ mother stock plants were cultivated under shade cloth (25% sunlight) in the field, Apopka, Florida, and were subjected to natural daylight. Plants were grown in 57 L containers filled with a soilless substrate, fertilized with controlled-release Osmocote fertilizer, and irrigated as described in Study I.
